# Frontal Conversion and Uniformity in 3D Printing by Photopolymerisation

**DOI:** 10.3390/ma9090760

**Published:** 2016-09-07

**Authors:** Alessandra Vitale, João T. Cabral

**Affiliations:** 1 Department of Chemical Engineering, Imperial College London, London SW7 2AZ, UK; 2 Department of Applied Science and Technology, Politecnico di Torino, Torino 10129, Italy

**Keywords:** 3D printing, photopolymerisation, conversion profile, photopolymerisation model, UV curing

## Abstract

We investigate the impact of the non-uniform spatio-temporal conversion, intrinsic to photopolymerisation, in the context of light-driven 3D printing of polymers. The polymerisation kinetics of a series of model acrylate and thiol-ene systems, both neat and doped with a light-absorbing dye, is investigated experimentally and analysed according to a descriptive coarse-grained model for photopolymerisation. In particular, we focus on the relative kinetics of polymerisation with those of 3D printing, by comparing the evolution of the position of the conversion profile (*z_f_*) to the sequential displacement of the object stage (*∆z*). After quantifying the characteristic sigmoidal monomer-to-polymer conversion of the various systems, with a combination of patterning experiments, FT-IR mapping, and modelling, we compute representative regimes for which *z_f_* is smaller, commensurate with, or larger than *∆z*. While non-monotonic conversion can be detrimental to 3D printing, for instance in causing differential shrinkage of inhomogeneity in material properties, we identify opportunities for facile fabrication of modulated materials in the *z*-direction (i.e., along the illuminated axis). Our simple framework and model, based on directly measured parameters, can thus be employed in photopolymerisation-based 3D printing, both in process optimisation and in the precise design of complex, internally stratified materials by coupling the *z*-stage displacement and frontal polymerisation kinetics.

## 1. Introduction

During the past decade, 3D printing has grown rapidly and the industry is expected to reach up to 20 billion USD by 2020 [1], as both manufacturing cost and, critically, time decrease. Current 3D applications include automotive, aerospace, medical and dental, as well as design, jewellery and increasingly domestic use. Numerous high-tech and specialist industries are actively piloting and already employing 3D printing technologies [2,3] in emerging applications in medical and analytical sciences [4], including tissue engineering [5,6,7], drug delivery [8], and reaction ware [9,10], but also device fabrication and microfluidics [11,12], materials for energy [13,14], low-density, high-strength materials [15,16], electronics [17,18,19].

3D printing is an additive manufacturing process in which successive layers of a material are patterned and combined to form 3D shapes. The process begins with the digitalisation of the model of the object to be produced and its subsequent slicing into model layers. Then, 2D layers are printed and added one on top of the other, forming the final product. 3D printing encompasses a large range of technologies that allows for processing different materials, including polymers, metals, ceramics, fibres, and nanocomposites [2]. For 3D patterning polymeric materials, extrusion- or melt-type techniques (e.g., fused deposition modelling, selective laser sintering) [20,21] are common methods for the fabrication of thermoplastic parts. However, these techniques have the drawback of comparatively low resolution, weak layer adhesion, and slow processing. Light-based technologies offer attractive routes for 3D printing of polymers and composites. Taking advantage of the exceptional spatial control and versatility of photopolymerisation reactions, such technologies can dramatically improve printing resolution and speed. Moreover, layer-to-layer interaction is significantly advanced, enhancing the mechanical properties of the printed objects. Examples of light-based 3D printing technologies include stereolithography (SLA), and digital light processing (DLP) [22,23]. In the former, specific surface regions of photosensitive liquid resin undergo localized polymerisation and crosslinking by exposure to a scanning spot light source (typically, a UV laser), while in the latter, all given portions of a layer are simultaneously photocured, significantly speeding up the process times.

UV-based 3D printing can be used with a wide variety of photosensitive monomers and resin systems and can adopt either a top-down (the light source irradiates the sample from the top, while the platform is lowered into the monomer bath during the process) or a bottom-up (the sample is irradiated from the bottom, as the inverted support platform is incrementally raised) geometry. Moreover, a process that utilises continuous rather than a stepwise building process, increasing significantly the part production speed and surface finish, has been recently developed [24].

In light-based 3D printing, lateral dimensions of each processed layer are set by the illumination system (light beam scanning or mask), whereas the patterned thickness (in the *z-*direction) is not defined by the film deposition (e.g., spin coating), as in conventional photolithography, but rather by the displacement of the support platform and the photopolymerisation reaction kinetics of the resin. These two stages generally happen sequentially: following a light exposure step of a prescribed dose, the solid-liquid interface is displaced away from the light source by a defined distance *∆z*. The nonlinear conversion of photopolymerisation and discrete displacements generally yield a non-monotonic spatial monomer-to-polymer conversion of the material. Moreover, fine pattern transfer required for high quality printing involves considerable light absorption, which intrinsically accentuates the conversion profiles of the 3D object layers. This non-homogeneity in photocuring conversion has potentially significant consequences for variations in network properties, such as refractive index, density, shrinkage, elastic modulus and permeability, and can be either detrimental or advantageous. On the one hand, it can lead to undesired surface roughness or even part failure, while on the other hand it can create desirable effects (e.g., controlled and periodic variation in refractive index, often employed in advanced and diffractive optics).

The rapid improvements in processing and the advances in materials used in light-induced 3D printing are expected to enable further customisation and complexity of manufactured parts. However, to ensure robust 3D printing processes and to fully exploit the intrinsic non-linearities of photopolymerisation, there is a clear need for investigating and controlling the spatio-temporal monomer-to-polymer conversion of the printed object in three dimensions.

This paper examines directly the evolution of network propagation and its consequence in terms of patterned height and network conversion in photopolymerisation, considering both experiments and theory underpinning the process. We report photocuring results for a range of acrylates and thiol-ene systems of various monomer architectures, and review practically useful modelling approaches that describe key features of photopolymerisation reaction, relevant for 3D printing. In particular, we focus on the tunable conversion profiles of 3D printed parts along their thickness (*z*-direction).

## 2. Results and Discussion

**Figure 1:**
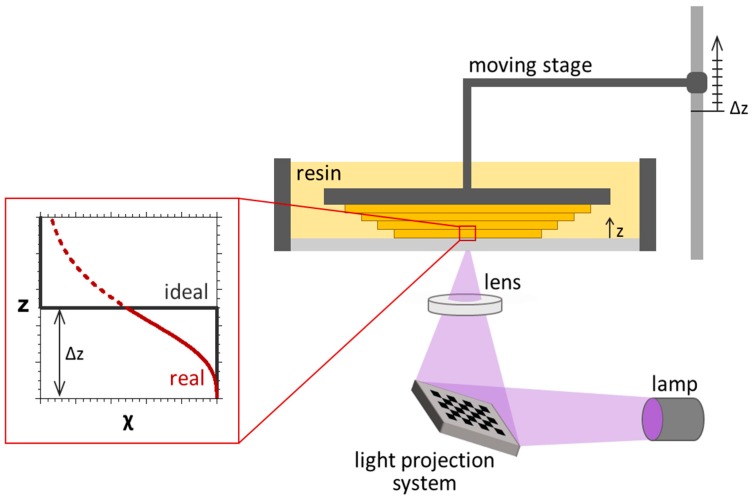
Schematic of a bottom-up 3D printer based on photopolymerisation. Each processed layer, with a thickness equal to *Δz*, is characterized by a non-constant monomer-to-polymer conversion *χ*, which shows a sigmoidal profile (**red**), and differs from the ideal square profile (**black**).

A representative bottom-up UV-3D printing system is depicted in Figure 1. It is mainly composed of a tank of photocurable resin, an illumination source (e.g., digital light projection system) and a platform, which is moved by a stepper motor and works as substrate for the growing object. The polymeric network is printed in a layer-by-layer manner and the height of the layers is controlled by the moving stage. After the first layer of material is photopolymerised and thus anchored to the platform, the stage is raised by a defined length (*Δz*), allowing a new layer of liquid resin to cover the surface of the object. This new layer is photopolymerised and the process continues sequentially with a series of *n* × *Δz* displacements, forming the 3D printed solid with the predetermined shape. Lateral shape and dimensions of patterning layers can be set by different techniques, such as digital imaging systems, photomasks, two-photon systems or rastered lasers, and the *xy* exposure of each distinct layer is modified as the *z* position incrementally evolves in the build process. The layer thickness (*z*-dimension) is thus defined by the displacement of the moving stage and corresponds to *Δz* (which generally is approximately 10–200 μm, depending on the required resolution, or surface roughness).

When the photocurable resin is irradiated, light-induced polymerization and crosslinking take place. Depending on the time of UV exposure and light intensity, a defined value of monomer-to-polymer conversion *χ* is obtained. The ideal conversion of each processed layer would be constant along *z*; however, *χ* is actually not uniform along the layer thickness and generally shows a propagating sigmoidal profile, illustrated in Figure 1, as it will be discussed below. Exceptional understanding and control of the spatio-temporal monomer-to-polymer conversion is required to manage and optimize the printing process and is thus the main purpose of this work.

The monomers used in photopolymerisation-based 3D printing should ideally be of relatively low viscosity, capable of rapid polymerisation yielding crosslinked polymers with properties suited to the demands imposed by the target application. Therefore, radical-mediated photopolymerisation reactions are generally employed: acrylates are most commonly encountered as photo-based printing materials [24], although vinyl-ether functionalized monomers (such as thiol-enes) are used as well [22,25]. In this study, we examine results obtained by photopolymerising and 3D printing a range of representative acrylate and thiol-ene photocurable systems.

Acrylate chemistry is suitable for light triggered 3D structuring due to the fast radical chain growth polymerisation that forms stiff networks within seconds. Generally acrylate monomers are preferred compared to methacrylates for their faster curing (although in some cases their brittleness can narrow their window of application). We select poly(ethylene glycol) diacrylate (diacrylate) as model acrylic system, since it is a biocompatible polymer generally used as biomaterial [26], and has already been adopted in 3D printing techniques [8,27,28].

**Figure 2:**
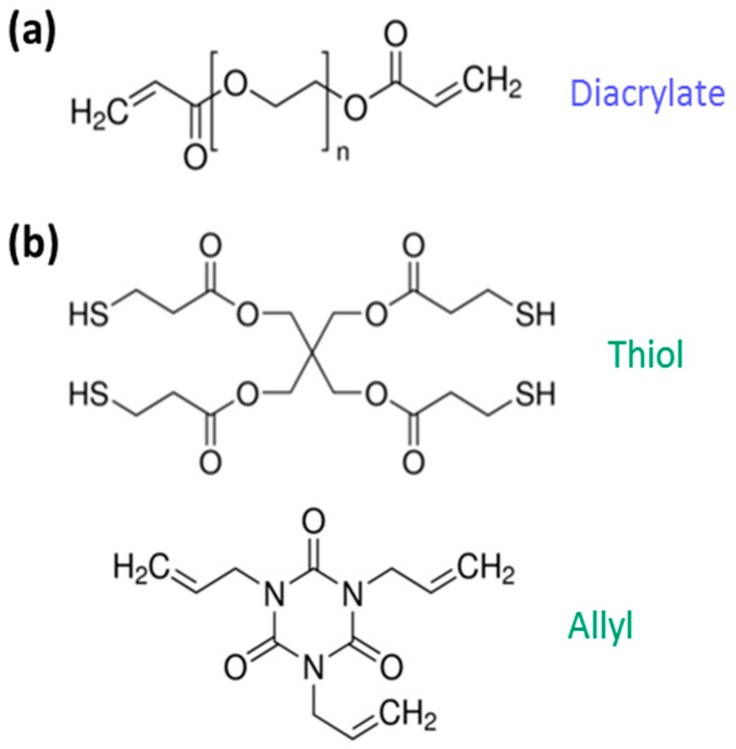
Chemical structure of model monomers used in this work: poly(ethylene glycol) diacrylate (diacrylate) (**a**); and thiol-allyl system (**b**), formed by 1,3,5-triallyl-1,3,5-triazine-2,4,6(1H,3H,5H)-trione and pentaerythritol tetrakis(3-mercaptopropionate) (these two monomers were mixed assuring an equimolar amount of allyl and thiol reactive groups).

Thiol-ene photopolymerisable formulations are interesting for the advancement of light-based 3D printing, as they form homogeneous networks via a radical step growth-like mechanism, show a delayed gelation and present a very limited polymerization induced shrinkage stress [29], which can be an issue for 3D printed pieces. As model thiol-ene systems, 1,3,5-triallyl-1,3,5-triazine-2,4,6(1H,3H,5H)-trione (allyl), mixed with pentaerythritol tetrakis(3-mercaptopropionate) (thiol), and NOA81, which is a commercial thiol-ene resin commonly employed as an adhesive and negative photoresist, were chosen. The chemical structures of the monomers used in this work are reported in Figure 2.

Because an ‘opaquing agent’ that absorbs UV light without reacting is generally used in UV-3D printing, in order to limit the depth to which the UV light can penetrate the resin solution and thereby ensure that a good *z*-resolution is obtained, a dye was added to selected formulations.

**Figure 3:**
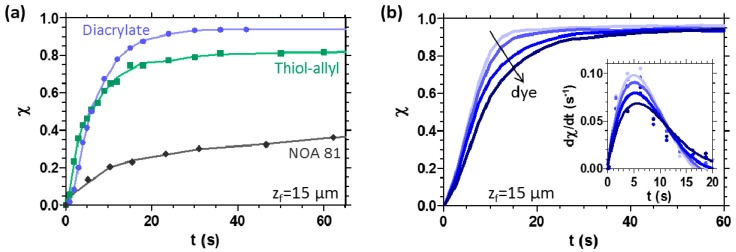
(**a**) Conversion *χ* as a function of exposure time *t*, for acrylic and thiol-ene systems, measured by FT-IR spectroscopy on 15 μm thick films, photopolymerised by using a light intensity *I* = 28 W·m^−2^; (**b**) influence of the dye on the photopolymerisation reaction kinetics, for diacrylate system (film thickness *z_f_* = 15 μm). The absorbing dye was added in the range 0–0.25 wt %. The inset reports the first derivative of the conversion, which is an indication of the reaction rate.

In light based 3D printing, the time *t* of each exposure step has to be judiciously selected depending on the chemistry of the resin, its absorbing properties and the thickness of the layer. In general, the irradiation time of each layer has to be sufficient to gel or solidify the material, forming a crosslinked network. The monomer-to-polymer conversion *χ* of the model systems, coated on a substrate as 15 μm thick films, was monitored by FT-IR spectroscopy and the photopolymerisation kinetics curves are reported in Figure 3a. These curves reproduce the typical behaviour of a multifunctional reactive system under irradiation, which gives rise to a three-dimensional solid network, and show that by increasing the irradiation time *t*, the photopolymerisation reaction progresses and the conversion of the resin advances. As clearly shown in Figure 3a, the kinetics of the reaction depends strongly on the chemistry of the system.

Furthermore, the addition of a dye affects the reaction kinetics (Figure 3b): as expected, the higher the concentration of dye, the slower the speed of the conversion reaction. Therefore, the dye and its concentration must be adequately selected based on the balance between decreased build times and enhanced *z*-resolution in a 3D printing process. The inset of Figure 3b shows the first derivative of the conversion curve, which represents the reaction rate, as a function of exposure time. The absorbing dye, depending on its concentration, affects both the polymerisation rate and the time required to reach the gel point, which can be defined as the conversion for which the maximum value of reaction rate is detected. An increase of the dye content is shown to cause a decrease of the maximum reaction rate and a delay in achieving the gel point.

The extent of polymerisation can also be expressed as *φ*, which is defined as the normalized conversion *χ* that can be measured experimentally, i.e., *φ*(*z*,*t*) ≡ *χ*(*z*,*t*)/*χ*_max_, where *χ*_max_ can be spectroscopically determined for each reactive mixture. In this context, the gel point of the photopolymerising system corresponds to a threshold value of conversion (*φ_c_*): below *φ_c_* the material is soluble, while above *φ_c_* a network is formed and the material results insoluble. It is thus analogous to a percolation threshold.

**Figure 4:**
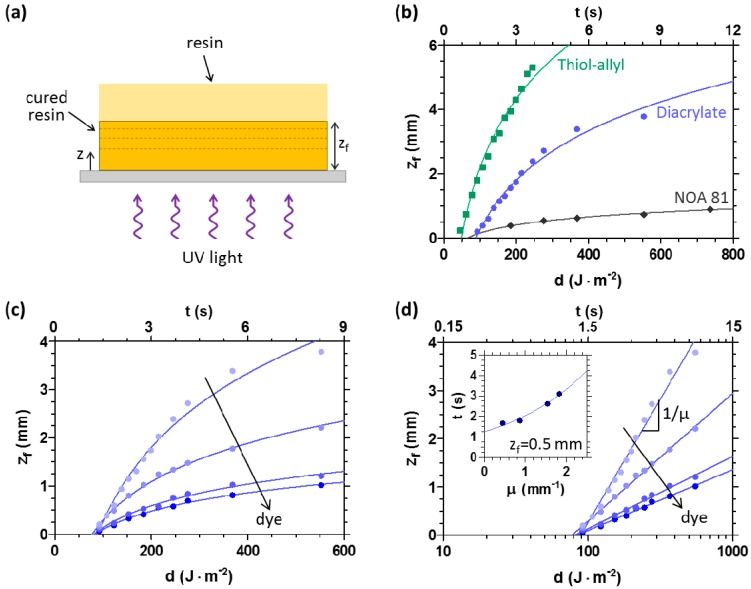
(**a**) Schematic of the FPP network formation in the ‘unbounded’ case (i.e., thick monomer layer), showing the front propagation from the UV illuminated surface. After a specific time (or dose) of UV exposure, the cured sample shows a well-defined thickness *z_f_*; (**b**) sample thickness *z_f_* dependence on UV exposure dose *d* and time *t*, for acrylic and thiol-ene systems. A UV light intensity of 67 W·m^−2^ was used; (**c**,**d**) effect of the introduction of a dye into the diacrylate system on the growth kinetics. The absorbing dye was added in the range 0–0.25 wt %, and the light intensity was 67 W·m^−2^. In (**d**), it is proven that the sample thickness *z_f_* increases logarithmically with *d* and *t*, after a critical value has been exceeded, showing a proportionality constant 1/*μ*. In the inset, the exponential dependence of the exposure time *t* required to polymerise a 0.5 mm thick sample on the optical attenuation coefficient *μ* for diacrylate system is reported.

As mentioned above, in UV-3D printing, the thickness of each layer is defined by the *z*-translation of the moving platform. However, it is important to consider that photopolymerising systems show also a well-defined ‘unbounded’ solidification thickness, when the liquid resin forms a thick layer (e.g., high *Δz*). This is an intrinsic property of the material resulting from the wavefront propagation kinetics of the solidifying network. In previous works [30,31,32], we have demonstrated that many acrylic and thiol-ene systems photopolymerise following a frontal photopolymerisation (FPP) process, characterised by the development and propagation of a travelling solidification wavefront into the monomer bath, driven by light. In the case of a thick resin bath (Figure 4a), the solidification front kinetics can thus be readily resolved by measuring the cured thickness *z_f_* following illumination and removal of the residual liquid monomer (i.e., development). Well-defined logarithmic growth kinetics in thickness with increasing exposure time *t*, or dose *d* (defined as the product of incident light intensity *I*_0_ and *t*) were established, as shown in Figure 4b. The exposure dose was found to precisely control front position and the process was well described by a minimal FPP model [30,31,33], summarised in Supplementary Information, capturing the non-linear spatio-temporal FPP growth. The front position (corresponding to the solidified thickness) reads:

(1) $$z_{f}\;=\;\frac{\ln\left[Kd/\ln\left(1/\left(1-\varphi_{c}\right)\right)\right]}{\mu }$$

where *K* is a material constant corresponding to an effective reaction conversion rate, *d* is the UV exposure dose (*d* ≡ *I*_0_ × *t*), *φ_c_* is the critical conversion *φ* threshold required to form a gel network (*z_f_* ≡ *z*(*φ* = *φ_c_*)), and *μ* is the optical attenuation coefficient of the material. The front position is therefore predicted to grow logarithmically with UV dose *d*, as experimentally confirmed in Figure 4b.

Furthermore, the inverse of this logarithmic slope is predicted to be *μ*, as demonstrated in Figure 4c,d, by adding an optically absorbing dye into the resin. Curing kinetics can thus be quantitatively tuned by adjusting *μ* via dye concentration. As a consequence, the time (or dose) required to obtain a UV cured sample with a defined thickness increases exponentially with the attenuation coefficient (see inset of Figure 4d).

The spatio-temporal evolution of the monomer-to-polymer conversion *φ* is predicted to be [31]:

(2)  φ (z,t) = 1 − exp[−Kdexp(−μz)].

**Figure 5:**
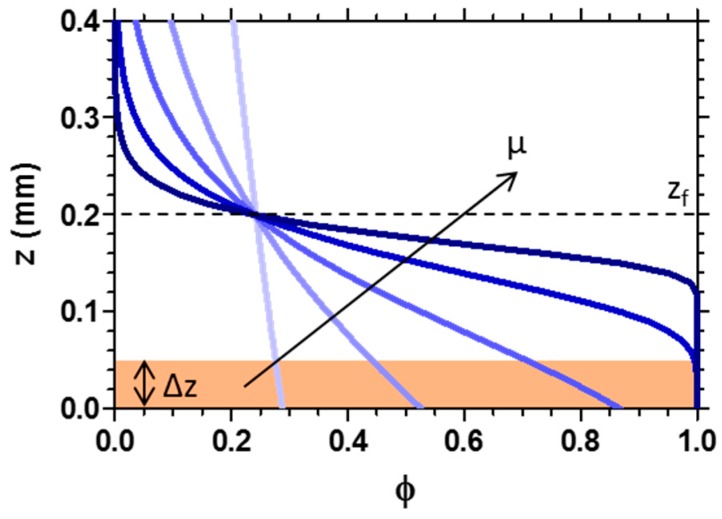
Conversion *φ* profiles along the sample depth *z* for increasing attenuation coefficient *μ*. The position of the photopolymerising front *z_f_* is fixed equal to 200 μm, and the printing layer thickness *Δz* is set as 50 μm.

The sigmoidal shape of the conversion profile (shown in Figure 5) remains time-invariant during propagation (see Figure S1 in the Supplementary Materials), in the absence of mass and thermal diffusion. The propagating front corresponds to the position and time where *φ*(*z*,*t*) = *φ_c_*. The profile described by Equation (2) is extremely important for UV-3D printing processes, as it provides quantitative insight into the conversion profile of each processed layer.

While this simple relation often holds in practice [32,34], the model can be extended to account for thermal effects [32,35], mass diffusion [36] and the changing of optical properties [32,33,36] during photopolymerisation. However, even in these cases, the conversion profile still maintains a *φ* sigmoidal shape, whose width and slope are strongly influenced by the absorbing properties of the photopolymerising material (Figure 5). High absorption (e.g., when a dye is added to the resin) intrinsically yields a steep non-monotonic spatial conversion. In 3D printing, geometries, and for systems (e.g., acrylates) where oxygen inhibition is important, the concentration of dissolved oxygen and its diffusion also needs to be considered [37], as well as its coupling with the polymerisation kinetics.

Evidently, the relative magnitude of the 3D printing layer thickness (i.e., *Δz*) and the position of the propagating front *z_f_* (as well as its interfacial width) is key in determining the resulting structure. Three cases can, in fact, be identified: (i) when *Δz* > *z_f_*, 3D printing fails, as each layer does not reach the threshold conversion (and thus does not form a network) along its length, and a new layer is cured on top the liquid ‘skin’ of the previous layer; (ii) when *Δz* ≈ *z_f_*, each photopolymerised layer can show a high variation of conversion along *z*; (iii) when *Δz* < *z_f_*, only small changes of *φ* are obtained along *z* in each processed layer (as illustrated in Figure 5). However, when the polymerisation front position *z_f_* per exposure is greater than the printing layer thickness *Δz*, the accurate printing of overhangs is clearly no longer possible.

**Figure 6:**
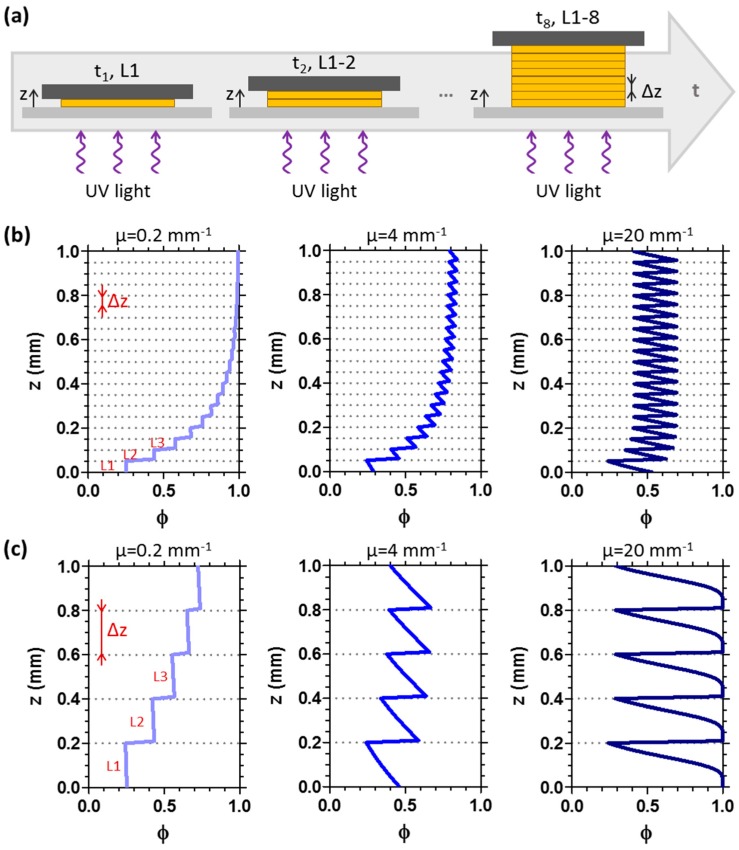
(**a**) Schematic of the step sequence during UV-3D printing. Each layer forming the printed object has a thickness corresponding to *Δz*. In (**b**,**c**), different conversion *φ* profiles along the sample depth *z* for 3D printed objects are reported. The layer thickness during the printing process (*Δz*) is set as 50 μm (**b**) and 200 μm (**c**). Profiles are modelled imposing *μ* = 0.2 mm^−1^, *μ* = 4 mm^−1^ and *μ* = 20 mm^−1^.

As depicted in Figure 6a, in 3D printing processes, a series of layers is polymerised in sequence with well-defined characteristics: initially, the first layer (L1) is UV irradiated for a defined time *t*; then, the displacement of the moving stage allows to cure the following layer (L2), and the process continues until the object reaches the desired thickness. As each layer has a thickness equal to *Δz*, the conversion *φ* along *z* of the printed object is the result of the sequential conversion of the building layers and hence exhibits a step-like profile (where each step has a length of *Δz*). A combination of material properties and process parameters thus controls the conversion profile of each layer and that of the entire object. While these parameters are generally optimised empirically to yield ‘good’ mechanical properties and surface roughness, we present a simple framework to enable the predictive design of 3D printed objects, with model parameters that can be readily measured experimentally. We emphasise that, in our view, understanding and controlling with precision the monomer-to-polymer conversion profiles is necessary to achieve the full potential of photopolymerisation in 3D patterning in both accelerating parameter selection and in developing novel printing approaches.

Examples of *φ* profiles that can be obtained by varying the *z*-displacement of the moving stage *Δz* and the optical attenuation coefficient *μ* of the resin are presented in Figure 6b,c. The values of *μ* selected to model the conversion profiles are typical for acrylic and thiol-ene photocurable systems [32], commercial photoresists [30,34] and formulations containing absorbing dyes [24] or fillers (e.g., SiO_2_, TiO_2_, carbon nanotubes) [31].

**Figure 7:**
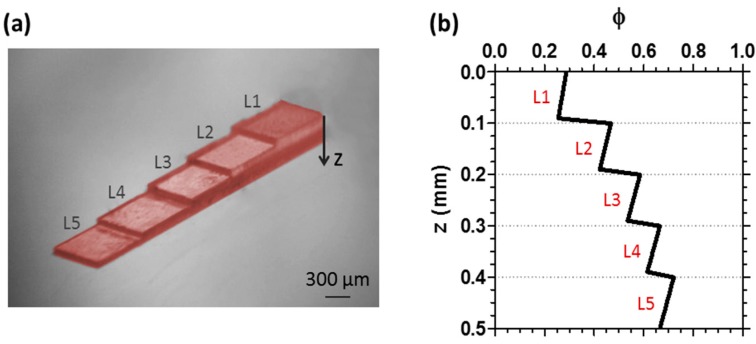
(**a**) Example of a UV cured sample made of coloured diacrylate, printed by setting 100 μm as single layer thickness; (**b**) profile of the step-gradient conversion *φ* along the sample depth *z*, modelled using the optical attenuation coefficient of the system (*μ* = 1.54 mm^−1^).

Figure 7 shows an experimental realisation of this approach, depicting a printed structure patterned from dyed diacrylate resin, in five 100 μm thick layers. For clarity, the *xy* illuminated area was varied at each exposure to highlight the distinct steps. The *φ* conversion profile, along the *z*-direction of the tallest section of this object (Figure 7b) shows a well-defined step-like gradient, demonstrating the simplicity of the approach. The stepped *z*-profile also illustrates how non-uniform conversion can also happen inadvertently with inevitable consequences in terms of shrinkage or modulation of material properties. Our coarse-grained model allows the facile prediction of these profiles.

Solidification occurs at UV doses well below full conversion and thus, while the object shape is set by the initial spatially-resolved exposure during 3D printing, conversion is completed by an intense, flood UV exposure termed ‘post-cure’. This stage serves multiple purposes, including raising the conversion of the polymer network, exhausting any residual initiator and enhancing the physico-chemical properties (e.g., the mechanical modulus) of 3D printed objects, thereby improving their stability. It is particularly important when using fast curing monomers and short irradiation exposures, desirable in 3D printing processes.

**Figure 8:**
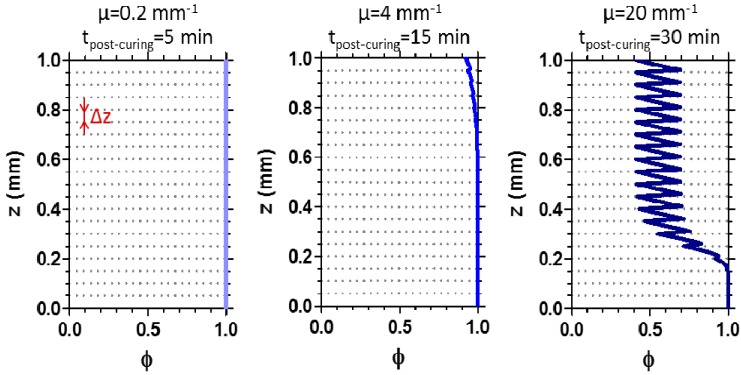
Conversion *φ* profiles along the sample depth *z* for 3D printed objects after post-curing. The layer thickness during the printing process (*Δz*) is set as 50 μm. Profiles are modeled imposing *μ* = 0.2 mm^−1^, *μ* = 4 mm^−1^ and *μ* = 20 mm^−1^, *I* = 3 W·m^−2^, and the post-curing time as 5 min, 15 min and 30 min, respectively.

Post-curing increases the uniformity of the conversion *φ* along the sample depth, i.e., across the different layers, as illustrated in Figure 8. Indeed, when the attenuation coefficient *μ* of the system is relatively small (e.g., *μ* = 0.2 mm^−1^), the stepped conversion profile can be completely eliminated by post-curing, thus yielding a printed object with uniform full conversion (*φ* = 1). When *μ* has an intermediate value (e.g., *μ* = 4 mm^−1^), post-curing strongly reduces the variation in conversion. In addition, the conversion profile can be further homogenized by increasing the post-curing time or light intensity, or by irradiating the sample from different directions. However, when the optical attenuation coefficient is high (e.g., *μ* = 20 mm^−1^), the post-curing process for a rather long time (30 min) enhances the conversion only on the surface closest to the light source, while leaving a significant periodic variation of *φ* in the other parts of the object.

The depth variation in *φ* can be both detrimental (e.g., leading to undesired surface roughness or failure) as well as advantageous. In fact, materials with tunable variations in network properties (e.g., refractive index, density, modulus, permeability) can potentially be useful for advanced applications. An example of such materials with interesting applications in optics and sensing, albeit generally at a smaller scale, is provided by distributed Bragg reflectors (DBRs), which are structures formed from multiple layers with periodic variation in the refractive index [38,39]. Our coarse-grained modelling, validated by a series of experiments, demonstrates the facile prediction and control of conversion profiles along the illuminated direction during photopolymerisation 3D printing, enabling the design of both uniform and structured 3D printed polymeric networks.

## 3. Materials and Methods

### 3.1. Materials

Poly(ethylene glycol) diacrylate (diacrylate) with a molecular weight of 700 g·mol^−1^, 1,3,5-triallyl-1,3,5-triazine-2,4,6(1H,3H,5H)-trione (allyl), and pentaerythritol tetrakis(3-mercaptopropionate) (thiol) were purchased from Sigma-Aldrich (Dorset, UK) and used as received. Thiol-allyl formulation was prepared with an equimolar amount of thiol and allyl reactive groups. Monomers were added to 1 wt % photoinitiator 2-hydroxy-2-methyl-1-phenyl-propan-1-one (Sigma-Aldrich) to obtain the final photocurable reactive mixtures. The thiol-ene based optical adhesive NOA81 was obtained from Norland Products (Cranbury, NJ, USA). Sudan I dye was purchased from Sigma-Aldrich and all other chemicals were obtained from VWR Chemicals (Lutterworth, UK).

### 3.2. Photopolymerisation Process

Photoymerisation was carried out at room temperature and atmospheric conditions, by means of a fiber optic UV lamp (Omnicure S1500, equipped with a 365 nm filter, Excelitas Technologies, Waltham, MA, USA). The reactive mixture was placed in a transparent vat and UV irradiated from the bottom for different exposure times *t* and using different light intensities *I*_0_. Between each irradiation step, a step-wise system was used to lift up the substrate, thus exposing fresh monomer to UV light. UV exposure was performed in the presence of a photomask, showing the inverse of the desired pattern geometry. Development of the patterned polymer networks was performed with appropriate selective solvents, acetone, ethanol or isopropanol, to remove uncrosslinked material. An inert atmosphere is not required for these experiments since the photoresist bath is confined by a glass substrate and oxygen inhibition (of the acrylate systems) is thus not significant. In some cases, a post-curing process was performed by irradiating the sample by means of a monochromatic 365 nm Spectroline SB-100P flood lamp (Spectronics, Westbury, NY, USA).

### 3.3. Characterisation

The thickness of the crosslinked samples was measured with a reflection optical microscope (Olympus BX41M, Southend on Sea, UK) and, for large thicknesses, with a digital caliper or a Dektak-XT stylus profiler (Bruker, Coventry, UK).

Photopolymerisation conversion and reaction kinetics were monitored by Fourier transform infrared (FT-IR) spectroscopy, using a Bruker Tensor 27 spectrometer coupled to a Hyperion microscope (Bruker, Coventry, UK). Thin films (i.e., about 15 μm) of the reactive monomeric mixtures were UV irradiated for defined times and FT-IR scans were acquired. The decrease of the area of the absorption band of reactive functionality (C=C acrylic groups or S–H thiol groups centred at 1640 cm^−1^ and 2570 cm^−1^, respectively) was observed. Absolute conversions *χ* were calculated using the ratio of peak areas to the peak area prior to polymerisation, employing the band at 1725 cm^−1^, assigned to C=O carbonyl groups, as reference:

(3) $$\chi \;=\;1\;-\;\frac{A_{t}/A_{t,\mathrm{ref}}}{A_{0}/A_{0,\mathrm{ref}}}$$

where *A_t_* and *A*_0_ indicate the area of the absorption band of reactive functionality at time *t* and at time *t* = 0 s, respectively; in our work, *A_t_*_,ref_ and *A*_0,ref_ indicate the area of the C=O peak at time *t* and at time *t* = 0 s, respectively.

The derivative of the first order of the conversion (d*χ*/d*t*) was calculated in order to evaluate the rate of photopolymerisation of the reactive mixtures. We defined the extent of polymerisation *φ* as the normalized conversion *χ* measured experimentally by FT-IR, i.e., *φ*(*z*,*t*) ≡ *χ*(*z*,*t*)/*χ_max_* for each polymeric system.

## 4. Conclusions

We investigate the spatio-temporal monomer-to-polymer conversion profile during photopolymerisation-based 3D printing processes. We experimentally examine the evolution of network propagation and the progression of the sigmoidal-shaped conversion during photopolymerisation for different acrylate and thiol-ene systems. In particular, we consider a diacrylate monomer (poly(ethylene glycol) diacrylate), a thiol-allyl system composed by a tetrafunctional thiol and a trifunctional allyl, and a transparent commercial resin based on thiol-ene chemistry.

Employing coarse-grained models that describe the photopolymerisation reaction, we demonstrate that the patterning process can be accurately predicted, both in terms of patterned height but also the conversion profile within the polymer network. This approach can thus be employed in the predictive optimisation of 3D printing parameters, which is currently done empirically, specifically the choice of exposure time and layer thickness *Δz*, as well as the formulation of the resin (minimally, monomer, photoinitiator and pigment). Our simplest model involves only two parameters, the light absorption coefficient *μ* and a rate constant *K*, in addition to a (fixed) conversion threshold for solidification *φ_c_*. The value of *φ_c_* (gel point) can be measured directly with IR spectroscopic analysis on the solid/liquid interface (i.e., the solidification front), or through a series of patterning experiments. The coefficient *μ* can be obtained from a single light absorption measurement of a slab of known thickness, or by the dependence of front position with time (or dose), as the inverse of the logarithmic slope. Finally, the rate constant can be calculated from the induction dose, which corresponds to the threshold UV dose required for the polymerising solid front to start propagating. These minimal parameters allow the computation of the conversion profile of a defined system, which is otherwise complex, to measure experimentally. The polymerisation conversion profiles can then also be tuned by the modification of printing parameters, such as the concentration of a light-absorbing dye or the UV dose. Because our method ensures the accurate control of the monomer-to-polymer conversion profile of each layer and that of the entire printed object, it has great potential for accelerating and optimising 3D printing processes. 

Moreover, we show that the relative magnitude of the thickness of 3D printed layers (*Δz*) and the position of the photopolymerising front (*z_f_*) is a key parameter in determining the resulting printed structure and its properties. We examine several regimes by comparing the 3D printing step *∆z* and the interfacial width to realise structures with internal conversion profiles, *φ* vs. thickness *z*, with a range from smooth to sawtooth patterns of well-defined profiles. The ability to design buried interfaces and stratified polymer materials with well-defined optical, mechanical, and shrinkage properties can be powerful for the production of advanced materials, including optically-active and shape-memory or self-folding (origami) materials.

## Supplementary Materials

The following are available online at www.mdpi.com/1996-1944/9/9/760/s1. Brief description of the FPP model, Figure S1. Conversion *φ* profiles along the sample depth *z* for increasing UV irradiation time *t*, which corresponds to an increasing sample thickness *z_f_*.
