# Supplementary material for: Frontal Conversion and Uniformity in 3D Printing by Photopolymerisation
Source: Materials (Basel). 2016 Sep 7;9(9):760. doi: 10.3390/ma9090760 (PMC5457111; doi:10.3390/ma9090760)
Supplement: Supplementary file 1 [file materials-09-00760-s001.pdf]

Supplementary Materials: Frontal Conversion and Uniformity in 3D Printing by Photopolymerisation

Alessandra Vitale and João T. Cabral

Brief Summary of the Modelling Approach

We model the spatio-temporal evolution of the photopolymerisation process [1–3] by introducing rate laws for the monomer-to-polymer conversion $\phi(z,t)$, and for the light intensity $I(z,t)$. The rate of change of $\phi(z,t)$ is taken to be proportional to the fraction of unconverted $(1 - \phi(z,t))$ and the light intensity $I(z,t)$ at the relevant depth z and time t, in addition to an overall reaction conversion rate K:

$$\partial\phi(z,t)/\partial t = K\,[1 - \phi(z,t)]\,I(z,t) \quad (1)$$

Upon photopolymerisation, the material comprises both converted and unconverted monomer, whose optical attenuation coefficients μ may differ. To account for this evolving optical attenuation, we write a generalized Beer-Lambert expression:

$$\partial I(z,t)/\partial z = -\bar{\mu}(z,t)\,I(z,t) \quad (2)$$

where the effective attenuation coefficient reads:

$$\bar{\mu}(z,t) \equiv \mu_0\,[1 - \phi(z,t)] + \mu_\infty\,\phi(z,t) \quad (3)$$

Corresponding to the neat attenuation coefficients of the unconverted and converted materials, weighted by their respective composition fractions, at a given time t and depth z. Evidently, in the limit of small variations in μ between monomer and polymer, the usual Beer-Lambert law is recovered:

$$I(z) = I(0)\,\exp(-\bar{\mu}z) \quad (4)$$

The main (analytical) solutions of this photoinvariant limit of our model are given in Equations (1) and (2) of the main paper. The spatio-temporal dependence of the conversion profile is plotted in Figure S1.

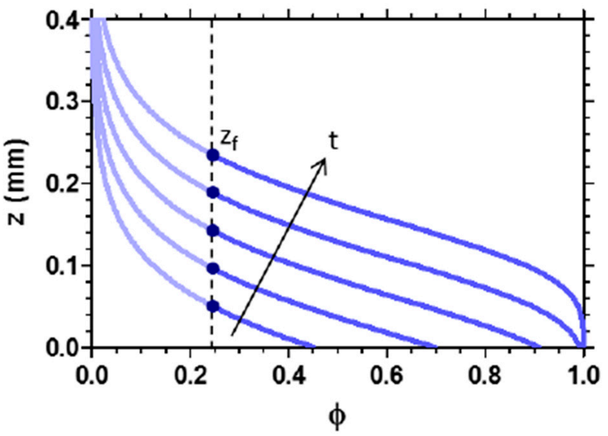

Figure S1. Conversion ϕ profiles along the sample depth z for increasing UV irradiation time t, which corresponds to an increasing sample thickness z_f.

In addition, an induction time is required for solidification, corresponding to the time interval associated to $\phi(z,t)$ first reaching the critical ϕ_c for solidification:

$$\tau \equiv \ln[1/(1 - \phi_c)]/KI_0 \quad (5)$$

and an equivalent expression can be written for the critical dose d_c.

The interfacial width W [3,4], characterizing the extent of the interface between the monomer and polymer network can be defined as:

$$W = \frac{1}{|d\phi/dz|_{\max}} \quad (6)$$

where $d\phi/dz$ is the maximum slope of the profile (at the inflection point), yielding $W = e/\mu$ and thus providing an effective means of estimating the steepness of the conversion profile. In the main paper, the interplay between this interfacial width and Δz of stereolithography is discussed in detail.

In a series of publications, the model has been extended to include mass diffusion [4] and heat generation and diffusion [5] during FPP, giving rise to a number of important corrections to our 'minimal model'. Nevertheless, the 'minimal model' reported above has been found to quantitatively describe a number of thiol-ene, both neat and filled systems, not only in terms of the kinetics of front position [1,2,6], but also the exact interfacial profile [6,7]. We have introduced a unified description for FPP that appears generally applicable to a wide range of free radical systems [7].

References

1. Cabral, J.T.; Hudson, S.D.; Harrison, C.; Douglas, J.F. Frontal photopolymerization for microfluidic applications. *Langmuir* **2004**, *20*, 10020–10029.
2. Cabral, J.T.; Douglas, J.F. Propagating waves of network formation induced by light. *Polymer* **2005**, *46*, 4230–4241.
3. Warren, J.A.; Cabral, J.T.; Douglas, J.F. Solution of a field theory model of frontal photopolymerization. *Phys. Rev. E* **2005**, *72*, 021801.
4. Hennessy, M.G.; Vitale, A.; Matar, O.K.; Cabral, J.T. Controlling frontal photopolymerization with optical attenuation and mass diffusion. *Phys. Rev. E* **2015**, *91*, 062402.
5. Hennessy, M.G.; Vitale, A.; Cabral, J.T.; Matar, O.K. Role of heat generation and thermal diffusion during frontal photopolymerization. *Phys. Rev. E* **2015**, *92*, 022403.
6. Vitale, A.; Hennessy, M.G.; Matar, O.K.; Cabral, J.T. Interfacial Profile and Propagation of Frontal Photopolymerization Waves. *Macromolecules* **2015**, *48*, 198–205.
7. Vitale, A.; Hennessy, M.G.; Matar, O.K.; Cabral, J.T. A Unified Approach for Patterning via Frontal Photopolymerization. *Adv. Mater.* **2015**, *27*, 6118–6124.
